# Drowning deaths between 1861 and 2000 in Victoria, Australia

**DOI:** 10.2471/BLT.16.174425

**Published:** 2016-11-21

**Authors:** Carolyn Staines, Joan Ozanne-Smith

**Affiliations:** a Department of Forensic Medicine, Monash University, 65 Kavanagh Street, Southbank, Victoria, 3006 Australia.

## Abstract

**Objective:**

To identify the long-term patterns of drowning mortality in the state of Victoria, Australia, and to describe the historical context in which the decrease occurred.

**Methods:**

We obtained data on drowning deaths and population statistics from the Australian Bureau of Statistics and its predecessors for the period 1861 to 2000. From these data, we calculated drowning death rates per 100 000 population per year, by gender and age. We reviewed primary and secondary historical resources, such as government and newspaper archives, books and the Internet, to identify changes or events in the state that may have affected drowning mortality.

**Findings:**

From 1861 to 2000, at least 18 070 people drowned in Victoria. Male drowning rates were higher than those for females in all years and for all ages. Both sexes experienced the highest drowning rate in 1863 (79.5 male deaths per 100 000 population and 18.8 female death per 100 000 population). The lowest drowning rate was documented in 2000 (1.4 male deaths per 100 000 population and 0.3 female deaths per 100 000 population). The reduction patterns of drowning mortality occurred within a historical context of factors that directly affected drowning mortality, such as the improvement in people’s water safety skills, or those that incidentally affected drowning mortality, like infrastructure development.

**Conclusion:**

We identified patterns of reduction in drowning mortality, both in males and females and across age groups. These patterns could be linked to events and factors that happened in Victoria during this period. These findings may have relevance to current developing communities.

## Résumé

**Objectif:**

Déterminer l'évolution sur le long terme de la mortalité imputable à la noyade dans l'État de Victoria, en Australie, et décrire le contexte historique dans lequel est survenue la diminution.

**Méthodes:**

Nous avons recueilli des données sur les décès par noyade et des statistiques démographiques auprès du Bureau australien des statistiques et des organismes qui l'ont précédé pour la période allant de 1861 à 2000. Ces données nous ont permis de calculer les taux de décès par noyade pour 100 000 individus par an, par sexe et par âge. Nous avons consulté des sources historiques primaires et secondaires, et notamment des archives publiques et de journaux ainsi que des ouvrages et des sites Internet, pour recenser les changements ou les évènements survenus dans l'État de Victoria qui ont pu avoir une incidence sur la mortalité imputable à la noyade.

**Résultats:**

On estime à au moins 18 070 le nombre d'individus morts par noyade entre 1861 et 2000 dans l'État de Victoria. Le pourcentage des hommes noyés était supérieur à celui des femmes quelle que soit l'année ou quel que soit l'âge des individus. Le taux de noyade le plus élevé, aussi bien pour les hommes que pour les femmes, a été calculé en 1863 (79,5 décès d'hommes pour 100 000 individus et 18,8 décès de femmes pour 100 000 individus). Le taux de noyade le plus bas a quant à lui été calculé en 2000 (1,4 décès d'hommes pour 100 000 individus et 0,3 décès de femmes pour 100 000 individus). La diminution de la mortalité imputable à la noyade est survenue dans un contexte historique de facteurs qui ont eu une incidence directe – comme l'amélioration des techniques de sécurité aquatiques des individus –, ou indirecte – comme le développement d'infrastructures – sur la mortalité imputable à la noyade.

**Conclusion:**

Nous avons mis en évidence la diminution de la mortalité imputable à la noyade aussi bien chez les hommes que chez les femmes et quelle que soit la tranche d'âge. Cette évolution peut être reliée à des évènements et à des facteurs intervenus dans l'État de Victoria pendant la période examinée. Ces conclusions peuvent se révéler utiles aux actuelles communautés en développement.

## Resumen

**Objetivo:**

Identificar los patrones de mortalidad por ahogamiento a largo plazo en el estado de Victoria, Australia, y describir el contexto histórico en el que se produjo la reducción.

**Métodos:**

Se obtuvieron datos acerca de las muertes por ahogamiento y estadísticas demográficas de la Oficina de Estadística de Australia y sus predecesores para el periodo comprendido entre 1861 y 2000. A partir de esos datos, se calculó la tasa de muertes por ahogamiento por cada 100 000 habitantes, por sexo y edad. Se analizaron fuentes históricas primarias y secundarias, como archivos gubernamentales y periodísticos, libros e Internet, para identificar cambios o acontecimientos acaecidos en el estado que pudieran haber afectado a la mortalidad por ahogamiento.

**Resultados:**

De 1861 a 2000, al menos 18 070 personas murieron por ahogamiento en Victoria. Las tasas de ahogamiento de los hombres fueron mayores que las de las mujeres en todos los años y en todas las edades. Ambos sexos experimentaron la mayor tasa de ahogamiento en 1863 (79,5 hombres fallecidos por cada 100 000 habitantes y 18,8 mujeres fallecidas por cada 100 000 habitantes). La tasa de ahogamiento más baja se documentó en el año 2000 (1,4 hombres fallecidos por cada 100 000 habitantes y 0,3 mujeres fallecidas por cada 100 000 habitantes). Los patrones de reducción de la mortalidad por ahogamiento se produjeron en un contexto histórico de factores que afectaron directamente a la mortalidad por ahogamiento, como la mejora de las medidas de seguridad acuática de las personas, o los que afectaron de forma incidental a la mortalidad por ahogamiento, como el desarrollo de infraestructuras.

**Conclusión:**

Se identificaron patrones de reducción en la mortalidad por ahogamiento, tanto en hombres como en mujeres y en todos los grupos de edades. Estos patrones podrían estar relacionados con acontecimientos y factores producidos en Victoria durante este periodo. Estos resultados pueden ser relevantes para las comunidades que actualmente se encuentran en desarrollo.

## ملخص

**الغرض:**

تحديد الأنماط طويلة الأمد للوفاة جرّاء الغرق في ولاية فيكتوريا في أستراليا، وتوضيح الظروف التي انخفضت فيها الوفاة جرّاء الغرق في الماضي.

**الطريقة:**

حصلنا على بيانات تتعلق بحالات الوفاة جرّاء الغرق وإحصاءات سكانية من المكتب الأسترالي للإحصاء والجهات المعنية السابقة له فيما يتعلق بالفترة من عام 1861 حتى عام 2000. وانطلاقًا من هذه البيانات، احتسبنا معدلات الوفاة جرّاء الغرق لكل 100000 نسمة في العام الواحد حسب النوع والعمر. وراجعنا المصادر الأساسية والثانوية المتعلقة بالفترة الماضية، مثل السجلات الحكومية وسجلات الصحف وبعض الكتب ومواقع الإنترنت، لتحديد التغيرات أو الأحداث التي شهدتها الولاية والتي قد تكون مؤثرة على حالات الوفاة جرّاء الغرق.

**النتائج:**

غرق 18070 شخص على الأقل في الفترة من عام 1861 حتى عام 2000 في ولاية فيكتوريا. وكانت معدلات الوفاة بين الذكور أعلى منها بين الإناث في جميع الأعوام ومن جميع الأعمار. وشهد الجنسان أعلى معدل للغرق في عام 1863 (79.5 حالة وفاة من الذكور بين كل 100000 نسمة و18.8 حالة وفاة من الإناث بين كل 100000 نسمة). وتم تسجيل أقل معدل للغرق في الوثائق في عام 2000 (1.4 حالة وفاة من الذكور بين كل 100000 نسمة و0.3 حالة وفاة من الإناث بين كل 100000 نسمة). وظهرت أنماط الانخفاض في حالات الوفاة جرّاء الغرق في ظل ظروف شهدتها الفترة الماضية ارتبطت بعوامل كان لها تأثير مباشر على حالات الوفاة جرّاء الغرق، مثل تحسين مستوى مهارات تحقيق السلامة عند التعامل مع البيئات المائية لدى الأشخاص، أو تلك العوامل التي أثرت على حالات الوفاة جرّاء الغرق دون التخطيط لذلك، مثل تطوير البنية التحتية.

**الاستنتاج:**

حددنا أنماطًا لانخفاض حالات الوفاة جرّاء الغرق، بين الذكور والإناث على حدٍ سواء مع النظر إلى الفئات العمرية. ويمكن الربط بين تلك الأنماط والأحداث والعوامل التي شهدتها ولاية فيكتوريا خلال الفترة المشار إليها. وقد ترتبط تلك النتائج بالمجتمعات النامية في الوقت الحالي.

## 摘要

**目的:**

旨在查明澳大利亚维多利亚州溺水死亡的长期模式，并描述死亡率降低的历史背景。

**方法:**

我们从澳大利亚统计局及其前身机构在 1861 年至 2000 年间的溺水死亡数据和人口统计数据。根据这些数据，我们按照性别和年龄计算出每年每 100 000 人中的溺水死亡率。 我们查阅了原始和次级历史资源，如政府和报纸档案、书籍以及网络，以查明可能影响该州溺水死亡率的变化或事件。

**发现:**

1861 年至 2000 年间，维多利亚州至少有 18 070 人死于溺水。 任何年份以及任何年龄段的男性溺水死亡率都要高于女性溺水死亡率。 两性溺水死亡率均在 1863 年达到最高值（每 100 000 人中有 79.5 位男性溺亡，每 100 000 人中有 18.8 位女性溺亡）。 根据文档记录显示，最低溺水死亡率发生在 2000 年（每 100 000 人中有 1.4 位男性溺亡，每 100 000 人中有 0.3 位女性溺亡）。 溺水死亡率的减少模式出现在直接影响溺水死亡率的因素得到改善的历史背景下，如人们的水上安全技能提高了，或者有意外影响溺水死亡率的因素，如基础设施建设。

**结论:**

我们查明了各年龄组男性和女性溺水死亡率的减少模式。 这些模式可能与维多利亚州当时所发生的事件和因素有关系。 该发现可能与当前社会发展有关。

## Резюме

**Цель:**

Определить долговременные тенденции смертности от утопления в штате Виктория, Австралия, и описать исторический контекст, в котором наблюдалось уменьшение этих случаев.

**Методы:**

Нами получены данные по смертям от утопления и статистическим показателям населения в Австралийском бюро статистики и в предшествующих ему организациях за период с 1861 по 2000 год. На основании этих данных нами были вычислены показатели смертности от утопления на 100 000 населения в год в зависимости от пола и возраста. Нами был проведен анализ первичных и вторичных исторических источников, таких как правительственные архивы и подшивки газет, а также книги и Интернет, с целью идентифицировать перемены или события в штате, которые могли затронуть смертность от утопления.

**Результаты:**

С 1861 по 2000 год в штате Виктория утонуло по меньшей мере 18 070 человек. Показатели утопления для мужчин были выше, чем для женщин, за все годы и во всех возрастных категориях. Наибольший показатель утопления для обоих полов был в 1863 году (смертность на 100 000 населения для мужчин составила 79,5, а для женщин — 18,8). Наименьший показатель утопления для обоих полов был в 2000 году (смертность на 100 000 населения для мужчин составила 1,4, а для женщин — 0,3). Уменьшение смертности от утопления происходило в историческом контексте, что определялось факторами, которые затрагивали смертность от утопления как непосредственно, например улучшение навыков людей по безопасности на воде, так и косвенно, например развитие инфраструктуры.

**Вывод:**

Нами определены тенденции уменьшения смертности от утопления среди мужчин и женщин и по возрастным группам. Эти тенденции могут быть связаны с событиями и факторами, которые имели место в штате Виктория в этот период. Эти данные могут иметь значение для развития общества в настоящее время.

## Introduction

Death due to drowning is a global problem. In 2012, an estimated 372 000 people drowned worldwide^1^ and the majority of such deaths occurred in low- and middle-income countries. The prevention of drowning deaths requires a complete understanding of the contributing factors, but the knowledge about drowning deaths in developing communities is sparse. Although epidemiological data have begun to emerge, they provide only a brief snapshot of the existing conditions. Some aspects of prevention, such as the development of water survival skills, for example swimming and rescue, or mechanisms to prevent drowning, for example the removal of hazards such as water-filled holes or the construction of barriers around hazards, require a fuller understanding of the changes in drowning patterns over time.

Injury prevention research has made little use of historical research methods.^2^ A few studies have examined drowning trends,^3^^–^^7^ but using relatively short time frames. One study has investigated flood-related drowning deaths between 1788 and 1996.^8^

Historical perspectives allow a depth of understanding that comes from seeing the phenomena in continuous and constant change and identifying the dynamics of that change.^9^ This study uses this perspective in a retrospective examination of long-term drowning death trends in a developing community, as the community evolved from exhibiting a high drowning risk to a low drowning risk.

In the financial year 2014–2015, the Australian state of Victoria had an unintentional drowning mortality rate of 0.67 deaths per 100 000 population.^10^ However, in 1905, Victoria’s drowning rate was 12 deaths per 100 000 population (Staines C and Ozanne-Smith J, Monash University, unpublished data, September 2006) resembling the rates in the World Health Organization (WHO) African Region (7.9 deaths per 100 000 population) and the WHO South-East Asia Region (7.4 deaths per 100 000 population) in 2012.^1^

Here we examine the changes in drowning incidence in Victoria between 1861 and 2000 and associate trends with historical factors. The results could be an epidemiological basis for a broader programme of research investigating the factors contributing to drowning deaths in this period.

## Methods

### Drowning mortality

We obtained drowning mortality and population data for Victoria from records of the Australian Bureau of Statistics and its predecessors for the period 1861 to 2000. No drowning mortality data were available for three years (1906, 1914 and 1931). The gender of those who drowned was available for all years except 1862 and 1917.

We calculated drowning death rates (deaths per 100 000 population per year) using either Australian Bureau of Statistics historical population data or estimates based on these data as the denominator.

To determine trend lines, we fitted structural time series models to the drowning data using the STAMP software package version 7.04 (Timberlake Consultants Ltd., London, United Kingdom of Great Britain and Northern Ireland).^11^

### Age

Because of variation in the reporting practices of the statistical agencies over time, age specific drowning data, and corresponding age specific population statistics, were available for only a limited number of years in the 19th and 20th centuries. We used the available data to determine drowning mortality patterns and age- and gender-specific drowning rates over four periods: mid-19th century, late-19th century, early-20th century, mid-20th century. The calendar years included in each period were for the mid-19th century: 1864 and 1866–1871; for the late-19th century: 1881–1885, 1895 and 1900; for early-20th century: 1901–1905, 1907–1913, 1915 and 1916; and for the mid-20th century: 1969–1978.

Drowning deaths were analysed in 5-year age groupings, in accordance with those employed by the Australian Bureau of Statistics.

### Historical context

To provide contextual information and to identify changes or events in the state that may have affected drowning mortality during the period of interest, we accessed a range of historical and contemporary resources. These included data from primary sources such as government department archives, primarily retrieved from Victoria’s Public Record Office and secondary sources, such as newspaper archives (available as either hard copies from newspaper and library archives or online sources, primarily the Australian online library database Trove). We also accessed historical and contemporary books and other literature from university or public libraries and electronic sources were accessed through the Internet.

## Results

From 1861 to 2000, at least 18 070 people died due to drowning in Victoria.

**Fig. 1:**
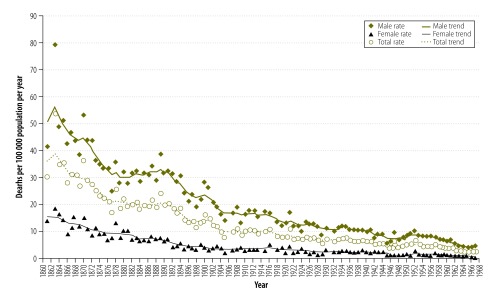
**Combined unintentional and undetermined drowning death rates and trend lines, Victoria, Australia, 1861–1967**

Note: The sample contained 15 698 drowning deaths.

**Fig. 2:**
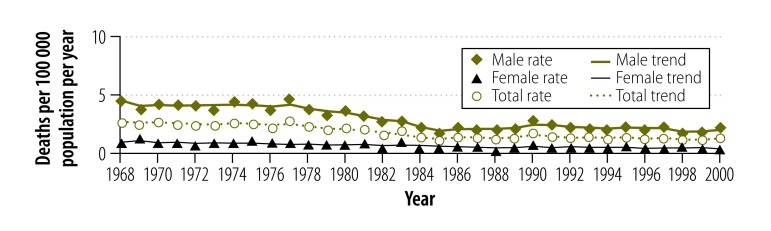
**Unintentional drowning death rates and trend lines, Victoria, Australia 1968–2000**

Note: The sample contained 2372 drowning deaths.

Before 1968, drowning statistics combined unintentional deaths with those where intent of death was undetermined. Therefore, the drowning death rates for the combined statistics (1861–1967) are shown in Fig. 1 and unintentional drowning death rates for the period 1968–2000 in Fig. 2. The overall pattern shows a reduction in drowning mortality, with total rates showing marked reductions from a maximum of 53.5 deaths per 100 000 population in 1863 to 0.8 deaths per 100 000 population by 2000. However, there were deviations from the general trend of reduction, there being some years of transient increase and periods of relatively little change.

A substantial proportion of the reduction occurred by the end of the 19th century. Based on 10-year averages, the total drowning rate decreased by 31.5%, from 34.3 to 23.5 per 100 000 population per year, by the 1880s and by 53.1%, to 16.1 per 100 000 population per year, by the end of the 19th century.

### Age

**Fig. 3:**
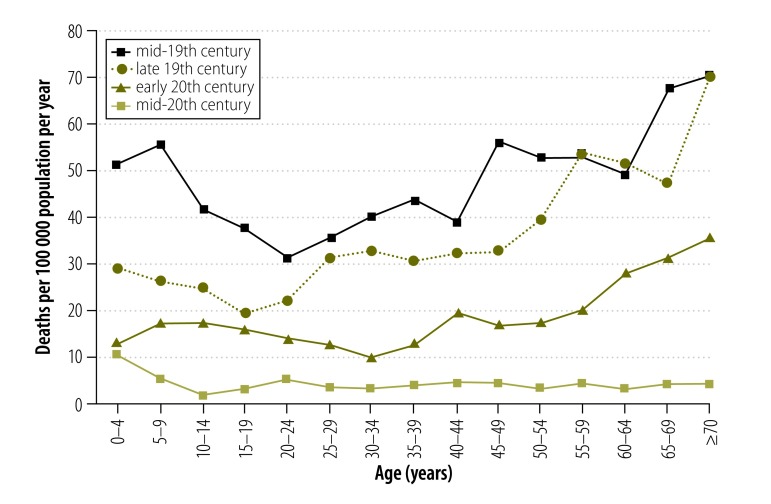
**Male drowning death rates, by age group and period, Victoria, Australia, 1864–1978**

Note: The calendar years included in each period were for the mid-19th century: 1864 and 1866–1871; for the late 19th century: 1881–1885, 1895 and 1900; for the early 20th century: 1901–1905, 1907–1913, 1915 and 1916; and for the mid-20th century: 1969–1978. The number of deaths included for the mid-19th century was 1169; for the late 19th century 1075; for the early 20th century1624; and for the mid-20th century 999.

**Fig. 4:**
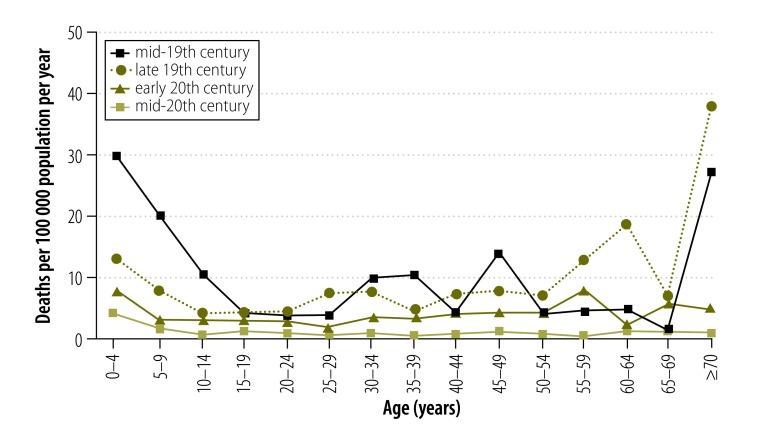
**Female drowning death rates, by age group and period, Victoria, Australia, 1864–1978**

Note: The calendar years included in each period were for the mid-19th century: 1864 and 1864–1871; for the late 19th century: 1881–1885, 1895 and 1900; for the early 20th century: 1901–1905, 1907–1913, 1915 and 1916; and for the mid-20th century: 1969–1978. The number of deaths included for the mid-19th century was 287; for the late 19th century 222; for the early 20th century 350; and for the mid-20th century 226.

**Fig. 5:**
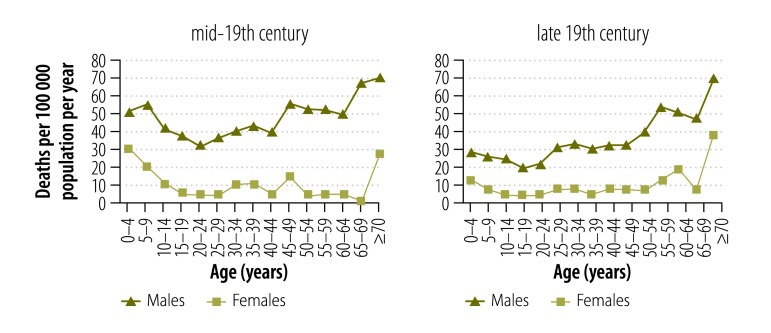
**Age-specific drowning death rates by period and gender, Victoria, Australia, 19th century**

Note: The calendar years included in each period were for the mid-19th century: 1864 and 1866–1871 and for the late 19th century: 1881–1885, 1895 and 1900.

**Fig. 6:**
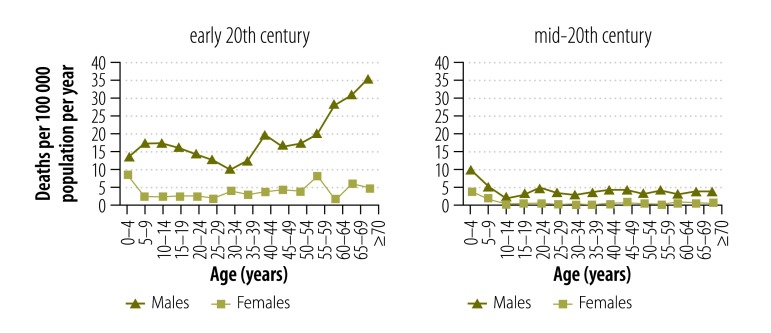
**Age-specific drowning death rates by period and gender, Victoria, Australia, 20th century**

Note: The calendar years included in each period were for the early 20th century: 1901–1905, 1907–1913, 1915 and 1916; and for the mid-20th century: 1969–1978.****

Fig. 3 shows age-specific drowning rates for males and Fig. 4 shows rates for females. Males aged 65 years or older had the highest drowning rates until the early-20th century. However, by the mid-20th century this had shifted to children younger than 5 years (Fig. 5 and Fig 6).

The drowning mortality rates in the mid-19th century were high for males aged 65 years or older and females aged 70 years or older, 65.6 deaths per 100 000 population and 32.6 deaths per 100 000 population, respectively. However, the proportions of total drowning deaths were small with only 1.7% (20/1169) of male and 1.4% (4/281) of female deaths. The pattern of high mortality rates for older people continued in the late-19th century for males and females aged 70 years or older, 66.6 deaths per 100 000 population and 30.3 deaths per 100 000 population, respectively, comprising 4% (43/1075) of male deaths and 5.6% (13/232) of female deaths.

While differing in magnitude, the drowning rates showed similar age-related patterns for males and females (Fig. 5 and Fig 6). Only in the early-20th century, were there marked differences between males and females in the patterns for the younger and older age groups.

### Sex

Both males and females showed reductions in drowning deaths over the period. For all years, the mortality rates for males were greater than for females. For both sexes, we observed the highest rates in 1863, 79.5 male deaths per 100 000 population and 18.8 female deaths per 100 000 population. The lowest rates were reported in 2000 with 1.4 male deaths per 100 000 population and 0.3 female deaths per 100 000 population.

#### Males

In the mid-19th century, all age groups had a very high drowning mortality of at least 31.3 male deaths per 100 000 population per year (20–24 year olds). The drowning mortality decreased in each subsequent era. Rates remained high until the early-20th century for all age groups (although unidentified intentional deaths may inflate the rates for this era). By the mid-20th century, only boys younger than 5 years had a high mortality, 9.4 deaths per 100 000 population per year (Fig. 3). There were several exceptions to the pattern of reduction. In the late-19th century, men aged 55 years or older continued to experience mortality rates resembling those of the previous era. Similarly, there was little change for 15–19 year olds from the late-19th century to the early-20th century and for boys younger than 5 years from the early-to mid-20th century.

The age-specific patterns for drowning mortality differed between the eras. For example, for males, the age group showing the lowest level of drowning mortality varied over time: 20–24 year olds in the mid-19th century; 15–19 year olds in the late-19th century; 30–34 year olds in the early 20th century; and 10–14 year olds in the mid-20th century (Fig. 3).

Boys younger than 5 years had high drowning mortality in all eras, with the mid-19th century showing the highest levels of mortality of 51.4 deaths per 100 000 population per year.

#### Females

The patterns of female drowning deaths are difficult to interpret. For most age groups there were so few cases that the patterns showed considerable variability. However, as with boys, girls younger than 5 years had relatively high levels of drowning mortality in all eras, with the mid-19th century showing the highest mortality (30.2 deaths per 100 000 population per year; Fig. 4).

### Historical context

**Fig. 7:**
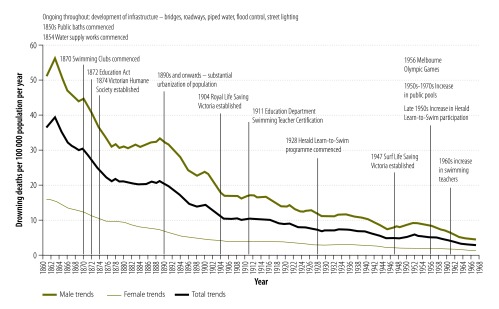
**Historical factors in relation to drowning death trends, Victoria, Australia, 1869–1967**

Note: The Herald Learn-to-Swim programme awarded achievement certificates to those, mainly school-aged children, who reached the target of swimming the distance of 25 yards (22.9 metres). It was conducted in conjunction with the Victorian Education Department and the life-saving societies. 

Fig. 7 shows a summary of the historical context of the drowning trends. In the 19th century, there were few coordinated efforts addressing drowning. While there were several individual factors that may have contributed to a reduction in drowning mortality, such as the establishment of bathing facilities providing safer venues for swimming and the commencement of swimming clubs, these did not show a synergistic effect until the 20th century. Much of the reduction in mortality in the 19th century was more likely due to the development of general infrastructure and the urbanization of the population.

#### Infrastructure development

Several aspects of the development of the physical infrastructure in the state could have affected drowning mortality. The development of a piped water supply system^12^ removed the need for reliance on hazardous water supplies or storage systems. Safe bridges and roadways, street lighting, the removal of water hazards, the construction of safety barriers, more substantial housing with perimeter fencing and the improved management of waterways would all be expected to have contributed to reductions in drowning mortality.

#### Urbanization

In the 1860s, the majority of residents lived in rural locations and 42% of people, including large numbers of children, lived in gold mining areas.^13^ By the 1880s, the population began to move away from rural locations, where they were exposed to many unprotected water hazards, and by the 1890s, almost half of the population was living in urban Melbourne.^13^

#### Other factors

The introduction of mandatory school attendance in 1872 could have contributed to a reduction in child drowning, by reducing the amount of time that children were unsupervised and in hazardous environments. In 1910, a coordinated programme for swimming and lifesaving instructions was established in state schools. The programme also included certification of swimming teachers, which increased the state’s capacity to provide children with water safety skills.

Much of the development of water survival skills in Victoria was associated with a supporting infrastructure of lifesaving agencies. In 1874, the Victorian Humane Society was established and while not having drowning prevention as its sole focus, it laid the basis for the later establishment of organizations whose primary aim was the prevention of drowning deaths. The Royal Life Saving Society Australia, Victoria Branch, took over the drowning prevention functions from the Victorian Humane Society in 1904 and in1947, the Surf Life Saving Society of Victoria joined this prevention effort. These two organizations have operated continuously in Victoria since their inception and have played important roles in the development of a culture of water safety, instruction in swimming, water safety and rescue and resuscitation skills. They also provide lifeguard services at recreational swimming venues.

One of the early barriers to developing water survival skills was the lack of safe swimming venues. However, in the mid-19th century, in response to the lack of private washing facilities in Melbourne, several public bathing houses were opened.^14^ While the initial focus was the improvement of hygiene, people started using the bathhouses for more recreational purposes and an interest in recreational bathing and swimming began to develop. In the 1870s, the first clubs for the promotion of swimming as a competitive sport were established. In the following century, hundreds of swimming clubs opened in urban and rural locations, which provided an infrastructure for population-wide exposure to water safety training.

The Games of the XVI Olympiad, held in Victoria in 1956, further led to the development of safe swimming venues. Building on the state’s strong interest in competitive swimming, the Olympic Games provided a further focus for the development of swimming infrastructure and resulted in a marked increase in the construction of Olympic sized swimming pools across the state.^15^

We also found that newspapers played a role in addressing the problem of drowning mortality. In the 19th century, newspapers reported coronial death inquests in considerable detail. The publications of drowning incident details could have contributed to an increased awareness of drowning hazards and may have generated an expectation in the community that authorities could and should do something to address the problems.

Newspapers continued to have a role in raising awareness throughout the 20th century. One metropolitan newspaper, in partnership with the education department and lifesaving agencies, established the state-wide Herald Learn-to-Swim programme in 1928.

## Discussion

Here we demonstrate that Victoria experienced a substantial reduction in drowning deaths between 1861 and 2000, both for males and females and across all ages. Contemporary drowning death statistics show that, worldwide, males experience higher drowning mortality than females.^16^ The results of this study concur with this pattern and that, in Victoria, the pattern has prevailed at least since 1861.

In this study, we used historical drowning statistics reported by the Australian Bureau of Statistics and its predecessors. Using historical statistics can be associated with problems due to changes in reporting practices. Before 1968, the Bureau aggregated unintentional drowning deaths and undetermined drowning deaths. Therefore, we reported the data separately for the years 1861–1967 and 1968–2000, since undetermined drowning deaths might have inflated mortality statistics before 1968. A review of a sample of undetermined deaths suggested that while 19th century statistics were relatively unaffected by undetermined deaths, early 20th century statistics may have included substantial proportions of these deaths.^17^ These findings suggest that one should interpret 20th century statistics before 1968 with caution.

We found very high levels of drowning mortality in children younger than 5 years in the mid-19th century. Studies done in contemporary developing communities have also reported similar or higher mortality levels. In Bangladesh, in 2003, drowning death rates for children younger than 5 years were reported to be 33.1 per 100 000 population in urban locations and 158.7 per 100 000 population in rural locations.^18^ In the Inner Mongolia Region of China, in 2012, children aged 1–4 years were found to have a drowning rate of 31.1 per 100 000 population.^19^

We also found that people aged 70 years or older had the highest drowning rates until the early-20th century. However, these results should be interpreted with caution, since the population size of this age group was small in this period and hence could have contributed to an overestimation of the mortality rates.

The mortality trends identified in this study show that while there is an overall decline in drowning mortality, the path is neither smooth nor steady. Some periods show a sharp decline, whereas others show an increase or no change. These changes in pattern are of potential interest, as they suggest that events might have happened that have induced these changes. Therefore, further investigating these changes would help to determine the possible underlying factors.

In Victoria, a substantial reduction in the number of drowning deaths occurred in the 19th century when there were few drowning prevention strategies in operation. Instead, mortality reduction at this time appeared to be an incidental outcome caused by several factors that indirectly resulted in a reduced exposure to hazards.

The implementation of specific drowning prevention strategies began in the late-19th century, but it appeared to take time before these strategies were working effectively. Coordinated and sustained drowning prevention strategies were not implemented until the 20th century. Following the establishment of life saving agencies, water skills’ programmes, community awareness strategies and regulatory interventions, drowning mortality continued to decline during the 20th century.

The patterns of drowning deaths and the associated historical context presented here might be of relevance to current developing communities that are interested in drowning prevention.
